# Δ^9^-Tetrahydrocannabinol Prevents Cardiovascular Dysfunction in STZ-Diabetic Wistar-Kyoto Rats

**DOI:** 10.1155/2017/7974149

**Published:** 2017-10-18

**Authors:** Rebecca K. Vella, Douglas J. Jackson, Andrew S. Fenning

**Affiliations:** School of Health, Medical and Applied Sciences, Central Queensland University, Rockhampton, QLD, Australia

## Abstract

The aim of this study was to determine if chronic, low-dose administration of a nonspecific cannabinoid receptor agonist could provide cardioprotective effects in a model of type I diabetes mellitus. Diabetes was induced in eight-week-old male Wistar-Kyoto rats via a single intravenous dose of streptozotocin (65 mg kg^−1^). Following the induction of diabetes, Δ^9^-tetrahydrocannabinol was administered via intraperitoneal injection (0.15 mg kg^−1^ day^−1^) for an eight-week period until the animals reached sixteen weeks of age. Upon completion of the treatment regime, assessments of vascular reactivity and left ventricular function and electrophysiology were made, as were serum markers of oxidative stress and lipid peroxidation. Δ^9^-Tetrahydrocannabinol administration to diabetic animals significantly reduced blood glucose concentrations and attenuated pathological changes in serum markers of oxidative stress and lipid peroxidation. Positive changes to biochemical indices in diabetic animals conferred improvements in myocardial and vascular function. This study demonstrates that chronic, low-dose administration of Δ^9^-tetrahydrocannabinol can elicit antihyperglycaemic and antioxidant effects in diabetic animals, leading to improvements in end organ function of the cardiovascular system. Implications from this study suggest that cannabinoid receptors may be a potential new target for the treatment of diabetes-induced cardiovascular disease.

## 1. Introduction

Diabetes mellitus is a disorder characterised by modifications in glucose homeostasis and ineffective secretion or action of insulin and, if not managed appropriately, leads to the development of hyperglycaemia [1]. Over a prolonged period, the hyperglycaemic state initiates chemical and metabolic changes to the myocardium and vasculature that manifest as diabetic cardiomyopathy and systemic vascular disease [2–5]. One of the major processes responsible for the development of the diabetic state and its pathological progression has been identified as the induction of oxidative stress [6].

Diabetes-induced oxidative stress is associated with overproduction of the reactive oxygen species superoxide by the mitochondrial electron transport chain and when produced in sufficient quantities it alters the body's redox balance [7]. Ultimately, the induction of oxidative stress leads to cellular damage as a result of increases in the flux of sugars through the polyol pathway, generation of advance glycation end products, activation of protein kinase C, and activity of the hexosamine pathway [7, 8]. Together with the direct pathological actions of reactive oxygen species, changes in cellular fuel handling also compromise function and mitochondrial respiration. These findings are supported by studies in diabetic humans and animal models where clinical use of novel antioxidant therapies restore redox balance translating to improved clinical end points [9–14].

A novel therapeutic target for the treatment of diabetes-induced cardiovascular disease is the cannabinoid receptors. Cannabinoid administration for medicinal purposes has been trialled in the treatment of multiple disorders including acquired immunodeficiency syndrome, multiple sclerosis, epilepsy, glaucoma, and Parkinson's disease [15] and initial cardioprotective effects have been demonstrated in an atherosclerotic murine model [16]. Cannabinoid administration to an animal model of type I diabetes has been reported to prevent the onset of oxidative stress within hepatic tissue [17], suggesting that its antioxidant effects may extend to the cardiovascular system as well. Recent reviews also summarise many of the cannabinoid-induced effects observed in the cardiovascular systems of animal models and humans and suggest cannabinoid receptors may be a promising new pharmacological target for the treatment of cardiometabolic disorders, provided that key clinical issues are addressed [18, 19].

The aim of this study was to determine if a nonspecific cannabinoid receptor agonist, THC, was capable of preventing alterations in cardiovascular function in an animal model of type I diabetes mellitus. By utilising a nonspecific cannabinoid receptor agonist in the present study it will provide an indication of whether cannabinoid receptors are viable targets for diabetes management and establish directions for future studies that can determine if any cardioprotective effects are linked to specific cannabinoid receptor subtypes.

## 2. Materials and Methods

### 2.1. Animals and Pharmaceutical Administration

Eighty, male Wistar-Kyoto rats were used in this study. Animals had unlimited access to water and food and were housed on a 12-hour light/darkness cycle. At eight weeks of age, animals were randomly assigned to one of four groups, Wistar-Kyoto controls (WKY, *n* = 20), WKY treated with THC (WKY + THC, *n* = 20), streptozotocin-induced type I diabetic animals (STZ, *n* = 20), and STZ treated with THC (STZ + THC, *n* = 20). THC was delivered via intraperitoneal injection (0.15 mg kg^−1^ day^−1^) for a period of 8 weeks. At the completion of the 8-week treatment period, animals were killed with an overdose of pentobarbitone (187.5 mg mL^−1^) delivered via intraperitoneal injection. Ethical clearance for this project was obtained from the Animal Ethics Committee of Central Queensland University under guidelines from the National Medical Research Council of Australia. All procedures conducted in this study were as humane as possible.

### 2.2. Induction of Diabetes

Diabetes was induced in 8-week-old male Wistar-Kyoto rats by a single intravenous injection of streptozotocin (65 mg kg^−1^) into the femoral vein, using a protocol previously outlined by Vella et al. [20]. Animals were considered diabetic when they displayed hyperglyceamia (>15 mmol L^−1^), polyuria, polydipsia, and a failure to thrive. Insulin was not administered to any animals during this study.

### 2.3. Biometric Assessment

Body mass and 24-hour water consumption were assessed once weekly for the duration of the study period. One day prior to euthanasia, systolic blood pressure and heart rate were assessed in lightly sedated animals (tiletamine 15 mg kg^−1^ with zolazepam 15 mg kg^−1^) using tail cuff plethysmography (ADInstruments, Bella Vista, Australia). At the time of euthanasia, blood glucose levels and wet mass of left ventricles, right ventricles, and kidneys, normalised to body mass, were recorded.

### 2.4. Biochemical Assessment

Serum levels of nitric oxide end products were assessed using a Griess reaction, as described by Cortas and Wakid, [21]. Serum malondialdehyde was measured via HPLC as outlined by Claeson et al. [22] and Sim et al., [23]. R&D Systems DuoSet ELISA Development System® was used for assessment of serum interleukin-1*β* and interleukin-6 concentrations.

### 2.5. Assessment of Vascular Function

As outlined by Vella et al. [20], thoracic aorta and second-order mesenteric arteries were suspended Tyrode's solution (all in mM concentrations: sodium chloride 136.9, potassium chloride 5.4, magnesium chloride 1.05, sodium dihydrogen orthophosphate 0.42, sodium bicarbonate 22.6, calcium chloride 1.8, glucose 5.5, ascorbic acid 0.28, and ethylenediaminetetraacetic acid 0.1) and gassed with oxygen (95%) and carbon dioxide (5%). Care was taken when threading vessels, to prevent damaging the endothelium. Once suspended in the experimental chamber, aortas and mesenteric arteries underwent a thirty-minute equilibration period, before being loaded with a preset resting tension of 10 mN and 2 mN, respectively. Vessels were then exposed to cumulative concentrations of noradrenaline, acetylcholine, and sodium nitroprusside and fluctuations to the preset tension after the addition of vasoactive agents were recorded using PowerLab® data acquisition units and LabChart software (ADInstruments, Bella Vista, Australia). Data is presented as concentration-response curves and percentage response curves and assessments of sensitivity and maximal response were generated for each agonist.

### 2.6. Assessment of Left Ventricular Function

Using a previously outlined protocol, left ventricular function was assessed using a Langendorff isolated perfused heart procedure [20]. Briefly, following euthanasia the heart was isolated and placed in ice-cold modified, gassed (95% oxygen and 5% carbon dioxide), Krebs-Henseleit Buffer (all in mM concentrations: sodium chloride 119.1, potassium chloride 4.75, magnesium chloride 1.19, potassium dihydrogen orthophosphate 1.19, sodium carbonate 25.0, glucose 11.0, and calcium chloride 2.16). The aorta was cannulated via the dorsal root and retrograde perfusion at a constant pressure of 100 mmHg was initiated. The apex of the heart was pierced to facilitate thebesian drainage and a latex balloon catheter connected to a pressure transducer was inserted into the left ventricle. Hearts were atrially paced at 250 beats per minute and measurements of end-diastolic pressure were recorded in 5 mmHg increments (beginning at 0 mmHg up to 30 mmHg) on LabChart software allowing for the assessment of diastolic stiffness (ADInstruments, Bella Vista, Australia). Maximal rates of contraction and relaxation were calculated at a load of 10 mmHg of pressure.

### 2.7. Assessment of Left Ventricular Electrophysiology

In accordance with Vella et al. [20], papillary muscles isolated from the left ventricle were suspended warm Tyrode's solution gassed with 95% oxygen and 5% carbon dioxide. Within the experimental chamber, two platinum electrodes were positioned on either side of the muscle and were connected to a bioamplifier. The muscle was then stretched to a preload of 5 mN. Field stimulation at a frequency of 1 Hz, a pulse width of 0.5 msec, and stimulus strength 20% above threshold was commenced. Following a ten-minute equilibration period intracellular bioelectrical activity was recorded using LabChart software (ADInstruments, Bella Vista, Australia). Parameters assessed included action potential duration at 20%, 50%, and 90% of repolarisation, resting membrane potential, and force of contraction.

### 2.8. Drugs and Chemicals

Reagents, chemicals, and drugs utilised in this study were of analytical grade and purchased from the Sigma Chemical Company and Thermofisher Scientific. The noradrenaline, acetylcholine, and sodium nitroprusside used in organ baths experiments were dissolved in distilled water. THC administered during the treatment regime was suspended in a solution of ethanol, Tween 80, and 0.9% saline to derive a preparation with a concentration of 0.15 mg mL^−1^ and streptozotocin for injection was suspended in citrate buffer immediately before use.

### 2.9. Data Analysis

Results were analysed on Prism software using two-way ANOVA with a Bonferroni post hoc test and Student's* t*-tests where appropriate. Significance was determined to be *P* < 0.05. All data are presented as mean ± standard error of the mean.

## 3. Results

### 3.1. Biometric Parameters: The Establishment of Diabetes and Its Impact on LV Haemodynamics

**Figure 1:**
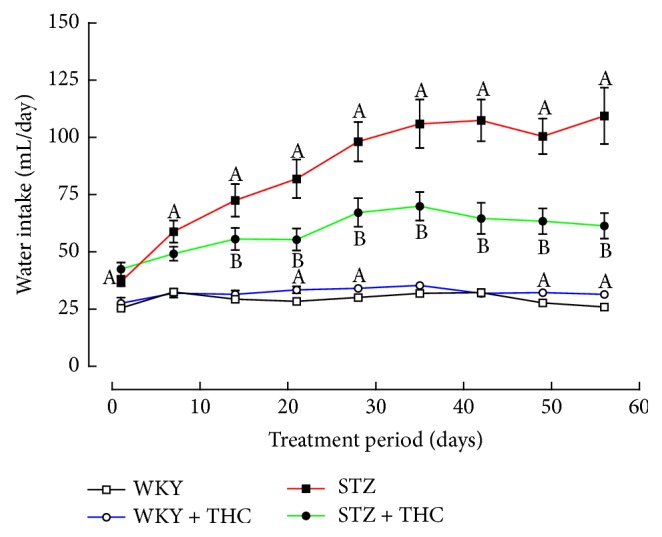
24-hour water consumption. All data are presented as mean ± standard error of the mean; ^A^*P* < 0.05 versus WKY and ^B^*P* < 0.05 versus STZ.

**Figure 2:**
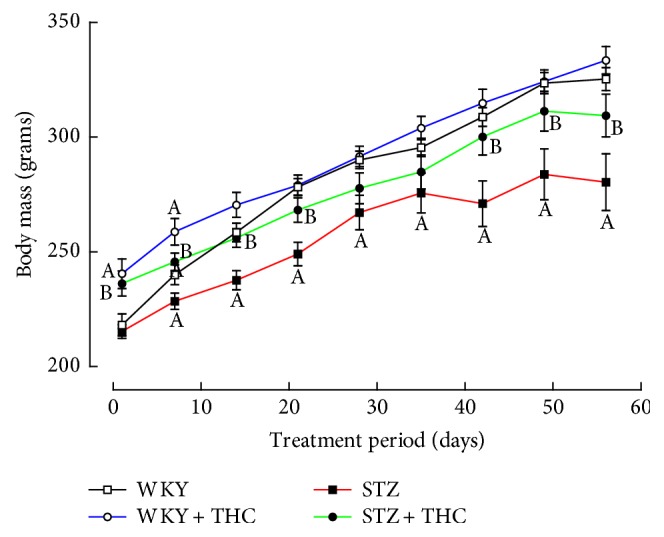
Weekly body mass. All data are presented as mean ± standard error of the mean; ^A^*P* < 0.05 versus WKY and ^B^*P* < 0.05 versus STZ.

Successful induction of diabetes was confirmed in STZ via significant increases in blood glucose concentration, increased water consumption, and an impaired ability to gain weight (Table 1 and Figures 1 and 2).

Cannabinoid administration significantly reduced hyperglycaemia in diabetic animals (21% reduction from STZ); however, blood glucose levels still remained 2.5 times higher than what was observed in WKY (Table 1). STZ + THC also consumed less water and had a moderate improvement in the ability to maintain body weight when compared to untreated diabetic group (Figure 1 and Table 1). The induction of diabetes did not alter systolic blood pressure but significantly reduced the heart rate of STZ (Table 1). THC administration to diabetic animals did not significantly impact these haemodynamic parameters (Table 1). Hypertrophy of the left and right ventricles was observed in diabetic rats (Table 1). Cannabinoid administration attenuated the development of left ventricular hypertrophy in STZ + THC (Table 1).

### 3.2. Biochemical Parameters: Cannabinoid Therapy Attenuates Diabetes-Induced Changes in Oxidative Stress

In comparison to WKY, the diabetic rats showed a 52% reduction in levels of nitric oxide end products and a significant increase in oxidative stress and lipid peroxidation via changes in serum malondialdehyde concentrations (Table 1). Diabetes-induced decreases in nitric oxide availability and increased circulating levels of malondialdehyde were attenuated by THC therapy in STZ + THC (38% increase in nitric oxide and 43% decrease in malondialdehyde) (Table 1). Diabetic rats showed no change in serum IL-1*β* concentration but had a significant reduction in serum IL-6 levels (Table 1). Cannabinoid therapy in diabetic animals significantly reduced serum IL-1*β* concentration and restored serum IL-6 concentration to levels similar to that observed in the WKY (Table 1).

### 3.3. Vascular Reactivity in Isolated Thoracic Aortic Rings: Cannabinoid Therapy Attenuates Diabetes-Induced Changes to Contractility and Relaxation of Conduit Vessels

**Figure 3:**
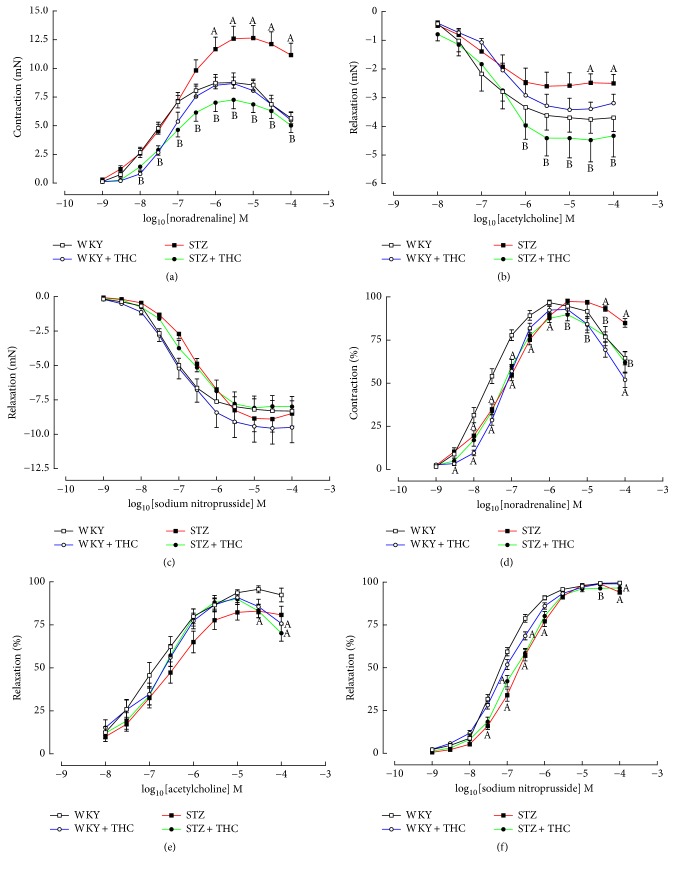
Cumulative concentration-response curves in thoracic aortic rings to (a) noradrenaline, (b) acetylcholine, and (c) sodium nitroprusside and percent maximal response curves in thoracic aortic rings to (d) noradrenaline, (e) acetylcholine, and (f) sodium nitroprusside. All data are presented as mean ± standard error of the mean; ^A^*P* < 0.05 versus WKY and ^B^*P* < 0.05 versus STZ.

Aortic tissue dissected from diabetic rats produced an increased noradrenergic contractile response and significant impairment of endothelial-dependent muscarinic induced relaxation (Figures 3(a) and 3(b)). These changes were accompanied by increased EC50s for noradrenaline and acetylcholine in the diabetic group (Figures 3(d) and 3(e) and Table 2). Cannabinoid therapy attenuated the increased noradrenergic contractility and impaired endothelial-dependent relaxation in diabetic animals but did not impact sensitivity of these agents to induce a response (Figures 3(a), 3(b), 3(d), and 3(e) and Table 2). The magnitude of endothelial-independent vasodilation was not affected by diabetes or THC therapy (Figure 3(c)); however, the EC50 for sodium nitroprusside was significantly increased in the tissue dissected from diabetic rats (Figures 3(c) and 3(f) and Table 2).

### 3.4. Vascular Reactivity in Isolated Mesenteric Arteries: Cannabinoid Therapy Attenuates Diabetes-Induced Changes in Endothelial-Dependent and Endothelial-Independent Relaxation of Mesenteric Arteries

**Figure 4:**
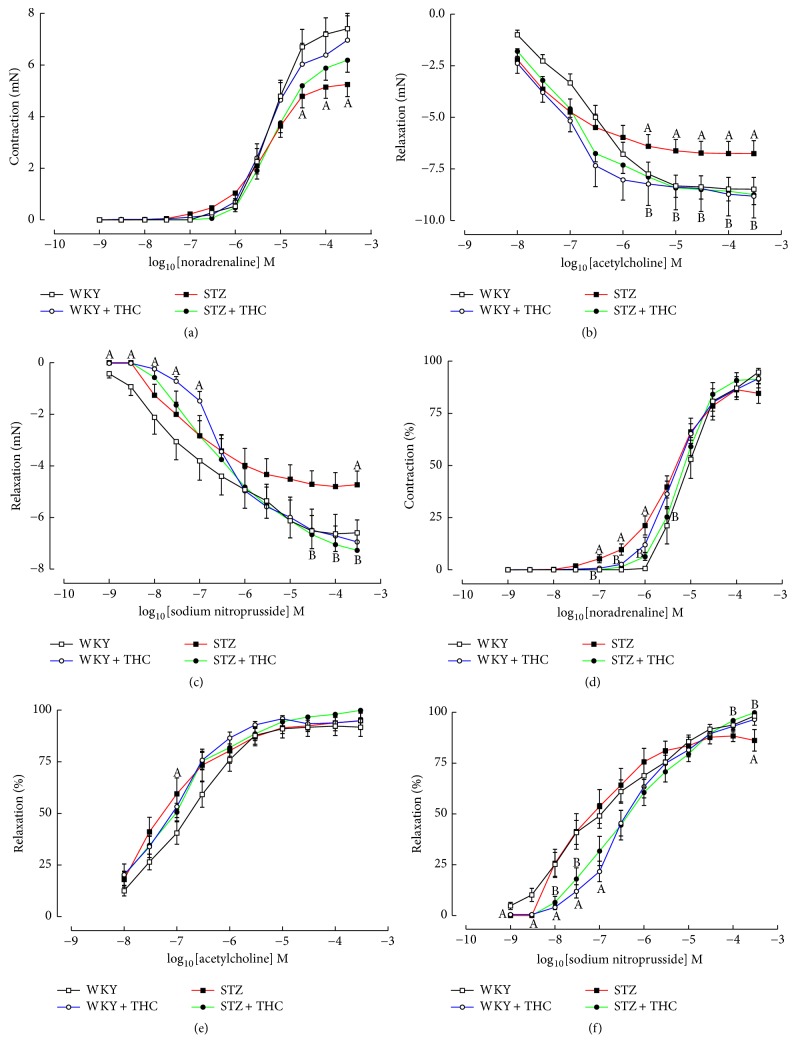
Cumulative concentration-response curves in mesenteric arteries to (a) noradrenaline, (b) acetylcholine, and (c) sodium nitroprusside and percent maximal response curves in mesenteric arteries to (d) noradrenaline, (e) acetylcholine, and (f) sodium nitroprusside; ^A^*P* < 0.05 versus WKY and ^B^*P* < 0.05 versus STZ.

Mesenteric vessels isolated from diabetic animals showed a reduced noradrenergic-mediated contractile response and reduced endothelial-dependent and endothelial-independent induced relaxation responses (Figures 4(a), 4(b), and 4(c)). These changes were accompanied by a reduction in EC50 for noradrenaline and acetylcholine (Figures 4(d) and 4(e) and Table 2). No significant alteration in the EC50 for sodium nitroprusside was observed in mesenteric tissue isolated from STZ (Figure 4(f) and Table 2). Cannabinoid therapy to diabetic rats significantly improved endothelial-dependent and endothelial-independent relaxation responses but did not prevent alterations in EC50 values for acetylcholine and sodium nitroprusside (Figures 4(b), 4(c), 4(e), and 4(f) and Table 2). Notably, the cannabinoid administration to STZ + THC significantly decreased the sensitivity of mesenteric vessels to sodium nitroprusside (Figure 4(f) and Table 2). Partial restoration of noradrenergic-mediated contractile response was observed in STZ + THC; however, this was not to a level of significance (Figure 4(a)). Cannabinoid administration to WKY was observed to increase sensitivity of resistance arteries to cholinergic mediated relaxation and decrease sensitivity of resistance arteries to noradrenergic-mediated contraction and sodium nitroprusside mediated relaxation, the latter of which was also observed in STZ tissue (Figures 4(d) and 4(e) and Table 2).

### 3.5. *Ex Vivo *Left Ventricular Function: Cannabinoid Therapy Attenuates Diabetes-Induced Changes in LV Compliance

Compared to WKY, the diabetic rats showed a significant increase in left ventricular diastolic stiffness (Table 3). THC therapy normalised the compromised left ventricular compliance observed in STZ + THC but did not significantly impact left ventricular functional indices (Table 3).

### 3.6. Left Ventricular Electrophysiology: Cannabinoid Therapy Attenuates Diabetes-Induced Prolongation of Cardiac Action Potentials

Left ventricular papillary muscles isolated from diabetic animals showed significantly prolonged cardiac action potential duration at 20%, 50%, and 90% of repolarisation and a significant reduction in force of contraction (Table 3). Cannabinoid administration to the diabetic rats attenuated prolongation of the action potential at 90% of repolarisation and significantly improved resting membrane potential and force of contraction (Table 3).

## 4. Discussion

Ineffective secretion or action of insulin leading to persistent hyperglycaemia is known to induce cardiovascular damage via oxidative stress and inflammatory mechanisms [2–6]. A key observation from this study was that cannabinoid administration to diabetic rats prevented alterations in markers of lipid peroxidation and oxidative stress, maintaining them at levels observed in control animals, and prevented maladaptive changes in the structure and function of the heart and blood vessels. These results indicate that, in an induced model of type I diabetes, activation of cannabinoid receptors may be a viable pharmacological target for the management of this metabolic disorder.

The THC-induced improvements in nitric oxide end products and lipid peroxidation observed in diabetic rats are similar to results reported in diabetic C57BL/6J mice, where administration of cannabidiol, for a period of 11 weeks, reduced intramyocardial accumulation of lipid peroxides, protein carbonyls, and reactive oxygen species [24]. Researchers have also shown that the binding site of anandamide, a cannabinoid structurally similar to THC, is coupled with nitric oxide release [25], suggesting a possible mechanism by which cannabinoids could induce NO bioavailability. Importantly, regulation of the redox state in STZ + THC conferred improvements in end organ function of the myocardium and vasculature. This is exemplified by preservation of myocardial pump function, cardiac electrophysiology, and noradrenergic-mediated contraction and endothelial-dependent relaxation of resistance arteries.

In addition to changes in redox state and lipid peroxidation, it is also possible that the THC-mediated cardioprotection reflects an improvement in the diabetic state, evidenced by a 21% reduction in blood glucose levels, when compared to STZ. Notably the administration of THC to STZ did not completely attenuate the diabetic state as blood glucose levels in these animals were still 2.5 times higher than controls. The impact of cannabinoids on blood glucose control is yet to be fully established and there are numerous and conflicting reports on the subject. The oral administration of a cannabinoid mixture (containing 4% THC) to STZ for eight days did not reduce blood glucose levels [17]. In comparison to results from the current study, this may suggest the composition of cannabinoid therapies and administration routes and also treatment length may be important in determining their effects on glycaemic control. Inversely, despite associations with increased caloric intake, marijuana use has been associated with lower body mass index and lower prevalence of diabetes mellitus; however, mechanisms underlying these two findings are yet to be established [26]. Recent studies have established cannabinoid receptors in metabolic tissues. Cannabinoid receptors have been identified in isolated pancreatic islet cells and activation of the CB_2_ receptor subtype is associated with improved glucose tolerance [27]. Therefore, further research should consider the use of cannabinoid receptor agonists, as manipulation of their receptors may offer an alternative treatment for metabolic disorders.

Reductions in blood glucose and the attenuation of imbalances in the redox state conferred myocardial- and vasoprotective effects in THC-treated diabetic animals. The impairment of vascular smooth muscle cells and reduction in endothelial cell function within the diabetic vasculature has been reported in aortic tissue from STZ and is associated with cellular changes that arise due to ineffective release of basal nitric oxide [25]. Reduced nitric oxide availability is an integral factor in the development of vascular dysfunction and known to induce structural changes to the endothelial and smooth muscle cells [28]. These changes are also linked to oxidative damage. The addition of angiotensin to isolated rodent vascular smooth muscle cells enhanced the production of superoxide, a reactive oxygen species known to function as a secondary messenger system to upregulate cellular hypertrophy [29]. In the current study, cannabinoid therapy attenuated modifications in functionality of conduit arteries to noradrenaline and acetylcholine and resistance vessels to acetylcholine and sodium nitroprusside. THC administration to diabetic animals was also observed to increase sensitivity of resistance vessels to acetylcholine and decreased sensitivity of resistance vessels to sodium nitroprusside. The latter two of these observations were also observed in control animals given cannabinoid therapy and warrant further research to identify mechanisms that underpin these effects. Notably the aforementioned vasoactive responses on vessel functionality that were elicited by cannabinoids have been associated with nitric oxide upregulation and cannabinoid receptor and peroxisome proliferator-activated receptors *γ* activation [30–33]. Similar upregulation of nitric oxide availability has also been attributed to improvements in vascular responsiveness in diabetic rats administered with ascorbic acid, vitamin E, and *α*-lipoic acid [34–36]. The reported THC-mediated increases in circulating nitric oxide are consistent with results from the current investigation and in conjunction with the observed reductions in lipid peroxidation and blood glucose concentration are associated with improved vessel function in diabetic animals.

The antioxidant and antihyperglycaemic actions of THC also afforded protection to the diabetic myocardium, as demonstrated by the attenuation of increased diastolic stiffness, increased ventricular hypertrophy, reduced force of contraction, and prolongation of the cardiac action potential. The initiating factors associated with these pathological endpoints have been identified as imbalances to the redox state, changes in myocardial metabolism, and increased lipid peroxidation leading to collagen deposition, cardiac hypertrophy, and alterations in cardiac ion channels [37–41]. Therefore, THC-mediated increases in nitric oxide end products and reductions in blood glucose concentration and malondialdehyde levels observed in the current study served cardioprotective effects evident via the improved left ventricular function. Similar observations have been demonstrated in studies investigating the administration of phytoestrogens and bioflavonoids on isolated ventricular myocytes and STZ, respectively [38, 42]. In the latter of these studies, quercetin and rutin limited ischaemia-reperfusion infarct size by attenuating lipid peroxidation in the diabetic myocardium [42]. Increasing nitric oxide availability and reducing oxidative stress in STZ via resveratrol administration has also shown to improve left ventricular pump function [43].

In addition to maintaining the structural integrity of the diabetic heart, the current study reports that THC prevented the prolongation of the cardiac action potential. Reductions in mean sinoatrial conduction time, sinus node recovery time, and atrioventricular node functional refractory period have been reported in humans following THC consumption [44] as has an increase in maximal rates of contraction and left ventricular pressure in spontaneously hypertensive animals following antagonism of cannabinoid receptors [45]. This latter study demonstrates that activation of the cannabinoid receptors has been attributed to cardioprotective effects; however, further research is required to determine if these protective effects are associated with antioxidant and antihyperglycaemic properties, or direct effects on ion channels, similar to WIN55,212-2-induced activation of inwardly rectifying potassium channels in rodent brains [46].

To this effect, when comparing results observed in diabetic and control animals given THC in the present study, there are notable differences, which may suggest the observed cardioprotective effects are related to rectifying the blood glucose imbalance. Notably in the present study, WKY + THC had significant increases in maximal rates of contraction and relaxation of the left ventricle, but no cardioprotective or pathological alterations in cardiac electrophysiology. Additionally there were no significant changes in the magnitude of contractile or relaxation responses in conduit or resistance arteries as observed in diabetic animals administered with cannabinoid therapy. Overall the disparity between physiological effects on the cardiovascular system of WKY + THC and SHR + THC suggests that the cardioprotective effects observed in the latter are likely attributed to improvement in the diabetic state. This also means that because the WKY + THC did not have a significant pathological increase in blood glucose concentration, similar cardiovascular outcomes would not be expected, especially if cannabinoid therapy were mediating its effects via rectifying blood glucose imbalances. All of this considered it should be noted that reductions in lipid peroxidation occurred in both diabetic and control animals in the present study, suggesting that cannabinoid therapy can serve antioxidant effects, independent of pathological levels of oxidative stress being present. Importantly further research is required to elucidate the mechanisms attributed to cannabinoid-mediated improvements in cardiovascular function that were observed in diabetic animals, and this comparison only serves as a potential hypothesis.

## 5. Conclusion

The aim of this study was to determine if a nonspecific cannabinoid receptor agonist could provide cardioprotective effects in a model of type I diabetes mellitus. Outcomes from this study indicate that THC administration to STZ improved functional parameters of cardiovascular health by reducing oxidative stress, lipid peroxidation, and blood glucose levels. These results indicate that activation of cannabinoid receptors may be a viable experimental target for the prevention of oxidative stress-induced complications in type I diabetes mellitus. In doing so, it has broadened the scope of future studies which should work to identify if THC-mediated effects in the diabetic state are attributed to activation of one or both of the cannabinoid receptors and to better understand the intracellular antioxidant mechanisms that are associated with ligand binding.

## Figures and Tables

**Table 1:** Physiological and biochemical parameters following THC administration to control and diabetic rats.

Parameter	WKY	WKY + THC	STZ	STZ + THC
Systolic blood pressure (mmHg)	149 ± 11	144 ± 8	133 ± 4	143 ± 5
Heart rate (beats per minute)	439 ± 4	409 ± 11^a^	359 ± 23^a^	353 ± 14^a^
Change in body mass (% change over treatment period)	51.6 ± 2.9	39.8 ± 3.5^a^	21.4 ± 3.4^a^	35.9 ± 4.5^b^
Left ventricular mass (mg gram body mass^−1^)	2.32 ± 0.03	2.43 ± 0.19	3.13 ± 0.15^a^	2.65 ± 0.09^b^
Right ventricular mass (mg gram body mass^−1^)	0.51 ± 0.02	0.56 ± 0.09	0.87 ± 0.09^a^	0.75 ± 0.05^a^
Kidney mass (mg gram body mass^−1^)	6.07 ± 0.12	6.17 ± 0.42	10.15 ± 0.41^a^	7.68 ± 0.32^b^
Blood glucose (mmol L^−1^)	9.8 ± 0.6	11.9 ± 0.8^a^	30.5 ± 1.6^a^	24.2 ± 2.3^b^
Nitric oxide end products (*µ*mol)	6.1 ± 0.5	4.6 ± 0.3	2.9 ± 0.2^a^	4.0 ± 0.2^b^
Serum Malondialdehyde (*µ*mol)	62.2 ± 2.7	13.4 ± 0.9^a^	94.3 ± 2.0^a^	53.7 ± 4.2^b^
Serum Interleukin-1*β* (ng mL^−1^)	4.8 ± 0.1	4.9 ± 0.1	4.7 ± 0.04	4.4 ± 0.02^b^
Serum Interleukin-6 (ng mL^−1^)	3.1 ± 0.1	3.1 ± 0.1	2.7 ± 0.1^a^	3.0 ± 0.03^b^

All data are presented as mean ± standard error of the mean; ^a^*P* < 0.05 versus WKY and ^b^*P* < 0.05 versus STZ.

**Table 2:** Vascular functionality in thoracic aortic rings and mesenteric arteries as assessed via EC50 and maximal response.

	Noradrenaline		Acetylcholine		Sodium nitroprusside	
	Log EC50	Maximal contraction	Log EC50	Maximal relaxation	Log EC50	Maximal relaxation
Thoracic aortic rings						
WKY	−7.58 ± 0.17	97 ± 2	−6.85 ± 0.04	96 ± 2	−7.14 ± 0.02	100 ± 0
WKY + THC	−7.11 ± 0.23	93 ± 2	−6.69 ± 0.15	91 ± 3	−7.00 ± 0.02^a^	99 ± 0
STZ	−7.15 ± 0.07^a^	97 ± 1	−6.37 ± 0.11^a^	83 ± 4^a^	−6.66 ± 0.03^a^	99 ± 0
STZ + THC	−7.12 ± 0.22	90 ± 4^b^	−6.65 ± 0.18	90 ± 3	−6.76 ± 0.02	97 ± 1^b^

Mesenteric arteries						
WKY	−5.02 ± 0.03	95 ± 2	−6.76 ± 0.06	92 ± 5	−6.88 ± 0.07	98 ± 1
WKY + THC	−5.21 ± 0.04^a^	92 ± 3	−7.13 ± 0.05^a^	96 ± 1	−6.26 ± 0.05^a^	97 ± 3
STZ	−5.27 ± 0.05^a^	86 ± 4	−7.17 ± 0.07^a^	95 ± 4	−6.94 ± 0.14	88 ± 3^a^
STZ + THC	−5.12 ± 0.03^b^	91 ± 4	−7.08 ± 0.04^a^	100 ± 0	−6.29 ± 0.05^b^	100 ± 0^b^

All data are presented as mean ± standard error of the mean; ^a^*P* < 0.05 versus WKY and ^b^*P* < 0.05 versus STZ.

**Table 3:** Left ventricular pump function and electrophysiological parameters following THC administration to control and diabetic rats.

Parameter	WKY	WKY + THC	STZ	STZ + THC
Action potential duration at 20% of repolarisation (msec)	18.7 ± 0.9	20.2 ± 0.7	21.8 ± 1.1^a^	19.4 ± 0.6
Action potential duration at 50% of repolarisation (msec)	28.7 ± 1.2	30.5 ± 2.0	34.1 ± 2.2^a^	31.0 ± 1.2
Action potential duration at 90% of repolarisation (msec)	58.8 ± 3.2	65.6 ± 6.8	102.4 ± 6.4^a^	82.7 ± 5.2^b^
Resting membrane potential (mV)	−65.0 ± 2.1	−71.7 ± 4.9	−58.0 ± 4.4	−75.0 ± 4.2^b^
Force of contraction (mN)	2.1 ± 0.4	2.4 ± 0.4	1.0 ± 0.3^a^	2.4 ± 0.4^b^
Diastolic stiffness	23.7 ± 0.7	23.3 ± 0.9	32.2 ± 1.0^a^	27.6 ± 1.2^b^
+*dP*/*dT*_max_ (mmHg sec^−1^)	1889 ± 68	2390 ± 56^a^	2288 ± 195	2390 ± 125^a^
−*dP*/*dT*_max_ (mmHg sec^−1^)	−1206 ± 97	−1547 ± 27^a^	−1438 ± 163	−1569 ± 125^a^

All data are presented as mean ± standard error of the mean; ^a^*P* < 0.05 versus WKY and ^b^*P* < 0.05 versus STZ.
